# Nutrient- and non-nutrient-based natural health product (NHP) use in adults with mood disorders: prevalence, characteristics and potential for exposure to adverse events

**DOI:** 10.1186/1472-6882-13-80

**Published:** 2013-04-09

**Authors:** Karen M Davison, Bonnie J Kaplan

**Affiliations:** 1 Department of Community Health Sciences, University of Calgary, Calgary, AB, Canada; 2 University of British Columbia, School of Population and Public Health, 2206 East Mall, Vancouver, BC V6T 1Z3, Canada; 3 Department of Paediatrics, University of Calgary, Calgary, AB, Canada

**Keywords:** Natural health products, Adverse events, Mood disorders

## Abstract

**Background:**

To address knowledge gaps regarding natural health product (NHP) usage in mental health populations, we examined their use in adults with mood disorders, and explored the potential for adverse events.

**Methods:**

Food and NHP intake was obtained from 97 adults with mood disorders. NHP data was used to compare prevalence with population norms (British Columbia Nutrition Survey; BCNS). Bivariate and regression analyses examined factors associated with NHP use. Assessment of potential adverse effects of NHP use was based on comparing nutrient intakes from food plus supplements with the *Dietary Reference Intakes* and by reviewing databases for reported adverse health effects.

**Results:**

Two-thirds (66%; 95% CI 56 to 75) were taking at least one NHP; 58% (95% CI 47 to 68) were taking NHPs in combination with psychiatric medications. The proportion of each type of NHP used was generally higher than the BCNS (range of p’s < 0.05 to 0.0001). When intakes from food and NHP sources were combined, a small proportion exceeded any Lowest-Observed-Adverse-Effect-Levels: only for niacin (n = 17) and magnesium (n = 6), two nutrients for which the potential for adverse effects is minimal. Conversely, about 38% (95% CI 28 to 49) of the sample were taking a non-nutrient based NHP for which previous adverse events had been documented.

**Conclusions:**

The prevalent use of NHPs in this population suggests that health care providers need to be knowledgeable about their characteristics. The efficacy and safety of NHPs in relation to mental health warrants further investigation.

## Background

People with mental health conditions often use a variety of therapeutic alternatives which are not necessarily disclosed to their primary health care provider. Remedies used may include natural health products (NHPs), defined as agents that include constituents such as vitamins and minerals, herbs, homeopathic medicines, traditional medicines, probiotics, and other products like amino acids and essential fatty acids [1].

National surveys of general populations have found that NHP use is increasing around the world [2-5], but few studies have examined individuals with mental health conditions. Available data suggest that NHP use tends to be higher in those with anxiety and depressive disorders [6-11]. From a public health perspective, there is some concern that NHPs may interact with conventional therapies. For example, studies have reported that St. John’s wort combined with trazodone, sertraline, or nefazodone may cause serotonin syndrome [12]. Reporting the potential for adverse health-related events such as these has gained increased attention globally as the information serves as a means to prevent harm to individuals and help formulate recommendations for health systems change [13]. Conversely, other investigations report beneficial effects of NHPs including lowered requirement doses of medications [14-17]. In either case, it would be useful to know more about NHP use in people with mental health conditions. This analysis examined characteristics of NHP use including potential for adverse events in adults with mood disorders.

## Methods

### Subjects, settings and procedures

Data from a cross-sectional nutrition survey of adults (> 18 years; n = 97) that were randomly selected from the Mood Disorders Association of British Columbia (MDABC) membership list and resided in the lower mainland of British Columbia (n = 1400) was used; the list was provided to researchers by the MDABC. Sample size verification was obtained using one-sample inference for a binomial proportion [18] with estimates of NHP use at 60% in the study population and general population use being 46% [19], alpha of 0.05, and statistical power of 80%.

Individuals (n = 146) randomly selected from the MDABC membership list were first sent a letter inviting their participation in the study. Subsequent to the invitation letter, a research staff member phoned the potential participant to ask if the research coordinator could contact them to discuss the project. Ten attempts were made to contact the potential participant if there continued to be no answer. Then the file was closed. For those who agreed to participate, the research coordinator reviewed procedures of the study over the phone including instructions of how to complete their food records that were mailed out to them. Overall, 97% of the 146 names drawn for the study were resolved; that is, they were located and contacted to participate. Of those, a range of 124 to 129 individuals were considered in-scope or eligible based on information available. Individuals were considered out-of-scope or not eligible if they were dead, had moved out of the area, or exhibited at least one of the exclusion criteria (e.g., pregnant, lactating, living in an institution). Of those contacted and eligible, 3% refused to participate in the study and 2% did not show up for their appointment (based on the upper bound of 129 eligible).

The study protocol including details about verification of mood disorder diagnosis (i.e., bipolar I, bipolar II, and depressive disorder) using the Structured Clinical Interview for DSM-IV Axis I Disorders [20] and Global Assessment of Functioning (GAF) [21], exclusion criteria (i.e., presence of another condition impacting mental health), current symptoms of depression and mania based on the Hamilton Depression Scale (Ham-D) [22], and Young Mania Rating Scale (YMRS) [23], and collection of nutrient intake data are detailed elsewhere [24]. Of those that participated in the screening to assess eligibility, two were found to be ineligible. The study’s protocol including consent procedures were approved by the University of Calgary’s Conjoint Health Research Ethics Board. The consent procedures involved first mailing the study’s consent forms to those who agreed to participate, so that they could review it at their leisure. Then when the participants attended their appointment, they first reviewed the consent forms with the research coordinator or clinical interviewer and signed the form once they fully understood all aspects of the study (i.e., all information would be held in strict confidence) and were agreeable to participating. Honourariums were provided to cover expenses (e.g., taxis, buses, gas, parking) and time associated with completing a 3-day food record and attending the office interview (about 180 minutes total).

As part of the face-to-face survey interviews conducted at the MDABC office, respondents brought all medications and natural health products (e.g., vitamin, mineral, herbal, botanical, and homeopathic preparations) they were taking. Details of NHP use based on type, composition (e.g., ingredients and amounts recorded from the labels), and frequency (e.g., taken daily, monthly) were taken by a trained interviewer.

### Statistical analysis

Nutrient and NHP intake data were collected using the standardized procedures of the British Columbia Nutrition Survey (BCNS) that included nutrient analysis of 3-day food records using ESHA software and the Canadian Nutrient File. Full information about the BCNS, nutrient analyses methods, and quality control procedures [25] are detailed elsewhere.

Comparisons of NHP use (e.g., prevalence) were made with the BCNS (population norm) and associated factors such as socio-demographic (i.e., sex, age, relationship status, education, income) and clinical (i.e., medication use, depressive versus bipolar disorder, GAF, years since diagnosis, BMI, psychiatric symptom scores) characteristics were analysed using binomial tests of two proportions, Fisher Exact statistics, Student t-tests, Mann–Whitney tests and correlations where appropriate. Variables that were found to be significant based on the bivariate analysis were then analyzed in the logistic (i.e., NHP use as dichotomous dependent variable) and multiple regression (i.e., number of NHPs used as continuous dependent variable) models to examine factors associated with NHP use. No more than four variables were placed into a model at one time and all models were evaluated to ensure they met statistical test assumptions (e.g., normality, homoscedasticity) and goodness of fit (e.g., graphical depictions of the residuals, Hosmer and Lemeshow’s goodness-of-fit test). All statistical analyses were conducted using Stata 7.0 software.

*Safety of NHP Use*: Nutrient content in NHPs was recorded for each participant, expressed as a daily amount and added to the usual intake obtained from food sources alone. For example, vitamin C from food sources averaged over 3 days was added to vitamin C from supplements expressed as a daily amount based on previous month’s use. Nutrient intakes (from food and NHPs combined) were compared to the Tolerable Upper Intake Levels (UL) of the *Dietary Reference Intakes* (*DRI*s), where applicable, to estimate prevalence of excess intakes. The ULs represent a daily nutrient amount for almost all *healthy* individuals where risk of adverse effects increases as intake levels exceed the standard [26]. For those intakes exceeding the ULs, the amounts were then compared to the Lowest Observed Adverse Effect Levels (LOAELs) of the *DRI*s, when available. The LOAEL is the lowest amount for which an adverse effect has been reported [26].

For NHPs without *DRI* comparison data, safety of use was examined by conducting detailed electronic searches of all products taken by participants for any reported adverse events. The databases searched included MEDLINE, EMBASE, PsychINFO, the Cochrane Library, CINAHL, NAPRALERT, MedEffect™ Canada, International Pharmaceutical Abstracts, CISCOM, and HerbMed. Search terms included common and scientific names, as well as synonyms for the NHPs and their primary active constituents. Adverse events included those reported from individual NHPs as well as any potential interactions that may have occurred from concurrent NHP use and with medications that participants were taking. We refer to this as the potential for adverse events previously defined as an unsafe state, not currently an event, but likely to lead to an event if it persists without intervention [27].

## Results

### Sample

The response rate was about 75% (97/129), calculated with the assumption that all the unresolved cases were in-scope (eligible). Those who did not want to participate in the study were asked a set of demographic health behaviour questions including their marital status, education level, use of vitamin and mineral supplements, type of bread and milk they consumed, and whether they smoked; no significant group differences were found between study participants and the non-responders based on these variables. The first author verified with the MDABC that the sample reflected the demographics (e.g., gender, age range) of their membership.

Most of the sample was female (n = 69; 71%; 95% CI 62% to 80%), had government-defined low income levels (n = 47; 49%; 95% CI 39% to 58%), had educational attainment levels less than a university degree (n = 76; 78.4%; 95% CI 70% to 87%), tended to carry excess weight (BMI > 25; n = 65; 67%; 95% CI 58% to 76%), had bipolar I or II disorder (60%; 95% CI 50% to 70%), and were considered to be high functioning based on mean GAF scores (62.7 ± 14.7) and median YMRS (Median = 3; 25^th^%ile = 1; 75^th^%ile = 5) and Ham-D scores (Median = 9.7, 25^th^%ile = 3.75; 75^th^%ile = 14.75).

### Prevalence and characteristics of NHP use

A total of 267 different NHPs were used in this sample. The proportion of the respondents taking at least one NHP was 66% (95% CI 56% to 75%). Fifty-eight percent of the sample (95% CI 47% to 68%) were taking NHPs in combination with their psychiatric medications that mainly included typical and atypical antipsychotics, antidepressants, and mood stabilizers; 8% (95% CI 4% to 16%) were taking these products without prescription medications. Of those who were taking NHPs, the average (mean) number of products used was 3 (range of 1 to 21; 95% CI 4 to 10). More than half of the sample were taking single or combination vitamin and mineral therapies, herbs and natural products and other nutrients such as glucosamine, amino acids, essential fatty acids from oils, and lactic acid bacteria (Table 1). Males had higher intakes of most single and combination preparations of vitamins, replacement preparations, and other nutrients (Table 1) (range of p’s < 0.05 to 0.0001). Comparisons of NHP use based on bivariate and regression statistical analyses with sociodemographic (i.e., age, education, income, relationship status) and clinical factors (i.e., psychiatric medication use, depressive vs. bipolar I and II disorder, GAF, years since diagnosis of mental health condition, BMI, depression scores, mania scores) showed no significant associations.

**Table 1:** Proportion taking NHPs in the previous month: comparison by sex and to the British Columbia Nutrition Survey (BCNS) (total sample = 97)

**NHP**	**Males (n = 28) % (95% CI)**	**Females (n = 69) % (95% CI)**	**Total (n = 97) % (95% CI)**	**BCNS (n = 1823) % (95% CI)**
**Water Soluble Vitamins (Single)**				
Vitamin B_6_ or pyridoxine	68 (51 to 85)^***^	14 (6 to 23)	31 (22 to 41)	0^a^
Vitamin B_9_ or folic acid	71 (55 to 88)^***^	28 (17 to 38)	40 (30 to 51)	0^a^
Vitamin C	64 (47 to 82)	67 (56 to 78)	66 (56 to 75)^+++^	24 (22 to 26)
**Fat Soluble Vitamins (Single)**				
Vitamin A	25 (9 to 41)^*^	4 (0 to 9)	10 (5 to 18)^+++^	2 (1 to 3)
Vitamin D	61 (43 to 79)	51 (40 to 62)	64 (54 to 79)^+++^	1 (0 to 2)
Vitamin E	46 (28 to 65)	46 (35 to 58)	47 (37 to 58)^+++^	17 (15 to 19)
**Vitamin Combinations**				
Vitamin B complex (with or without vitamin C)	68 (51 to 85)	49 (37 to 61)	55 (44 to 65)^+++^	9 (8 to 10)
Vitamin A and D combination	21 (6 to 37)^*^	4 (0 to 11)	9 (4 to 17)^++^	3 (2 to 4)
Multivitamins	14 (1 to 27)	19 (10 to 28)	18 (11 to 27)^+^	9 (8 to 10)
**Minerals (Single and Combinations)**				
Iron preparations	21 (6 to 37)	20 (11 to 30)	34 (25 to 44)^+++^	1 (0 to 2)
Single^b^ and multiple minerals^c^	79 (63 to 94)	78 (69 to 87)	78 (69 to 86)^+++^	13 (11 to 15)
**Vitamin and Mineral Combinations**				
Vitamins and minerals	61 (43 to 79)	59 (49 to 71)	60 (49 to 70)^+++^	31 (29 to 33)
**Other NHPs**				
Enzymes or gastrointestinal products^e^	46 (28 to 65)	30 (20 to 41)	35 (26 to 45)^+++^	6 (5 to 7)^d^
Replacement^f^ or homeopathic preparations^g^	46 (28 to 65)	29 (18 to 37)	34 (25 to 44)^+++^	5 (4 to 6)
Herbal and natural products^h^	61 (43 to 79)	51 (39 to 63)	54 (43 to 64)^+++^	19 (17 to 21)
Other products^i^	61 (43 to 79)^*^	88 (81 to 96)	75 (65 to 83)^+++^	20 (18 to 22)

^a^Pyridoxine and folic acid could not be analyzed statistically due to 0% prevalence in the BCNS.

^b^Includes chromium, selenium, zinc, and magnesium.

^c^Includes calcium plus magnesium; calcium, magnesium plus zinc; mineral combinations including bromide, calcium, silicon, nitrogen, selenium, phosphorous, iodide, chromium, manganese, titanium, rubidium, cobalt, copper, antimony, molybdenum, strontium, zinc, nickel, tungsten, germanium, scandium, vanadium, tellurium, tin, lanthanum, yttrium, silver, gallium, bismuth, zirconium, cerium, cesium, gold, beryllium, hafnium, samarium, terbium, europium, gadolinium, dysprosium, thorium, holmium, lutetium, erbium, ytterbium, neodymium, praseodymium, niobium, tantalum, thallium, rhenium, indium, and palladium.

^d^BCNS proportion < 1% but rounded to 1%.

^e^Includes antacids, adsorbents, laxatives, digestants.

^f^Electrolyte-type supplements intended to prevent or treat electrolyte imbalances that include sports drinks, over-the-counter powders and tablets, over-the-counter electrolyte replenishers, oral rehydration formulae, and multiple electrolyte injections. Most preparations contained sodium, potassium magnesium, and calcium.

^g^Homeopathic preparations contain medicinal ingredients and are prepared in accordance with the methods outlined in homeopathic pharmacopoeias.

^h^Includes herbs, herbal materials, herbal preparations and finished herbal products, that contain as active ingredients parts of plants, or other plant materials, or combinations.

^i^Includes glucosamine, amino acids, evening primrose oil, coenzyme Q10, flax seed oil, lactic acid bacteria.

Significant differences between males and females at ^*^p < 0.05, ^**^p < 0.001, and ^***^p < 0.0001.

Significant differences between study sample and BCNS at ^+^p < 0.05, ^++^p < 0.001, and ^+++^p < 0.0001.

### NHPs and potential for adverse events

When vitamin and mineral intakes from food and supplements were combined and compared to the *DRI*s, 2% (95% CI 0.3 to 7) to 8% (95% CI 4 to 16) of the sample had intakes that exceeded the ULs for 8 nutrients (Table 2); of these, 19% (95% CI 11 to 28) had niacin intakes above the LOAEL of 50 mg, and 6% (95% CI 2 to 13) had magnesium intakes above the LOAEL of 360 mg. In addition to the NHPs that had *DRI* comparison data, 16 other products used by 38% (95% CI 28 to 49) of the total sample were identified that could potentially lead to adverse effects as described in Table 3. The evidence cited in Table 3 often represents anecdotal reports of incidents that ranged from mild gastrointestinal discomfort to significant clinical events including worsening of mental symptoms.

**Table 2:** **Potential adverse events of vitamin and mineral supplement use based on comparison of nutrient levels to *Dietary Reference Intakes *and database searches for reported adverse events for individual vitamins and minerals used**

**Vitamin/mineral**	**UL**^**a**^	**% > UL**^**a **^**(95% CI)**	**LOAEL**^**b**^	**% > LOAEL**^**b **^**(95% CI)**	**Effect at the LOAEL**^**b**^
**Vitamins and Minerals with ULs**^a^**and LOAELs**^b^					
Vitamin B_3_ or niacin - mg	35^c^	28 (19 to 38)	50	19 (11 to 28)	Vasodilation causing flushing of the skin
Vitamin B_6_ or pyridoxine - mg	100	8 (4 to 16)	--	--	--
Vitamin B_9_ or folate - mcg	1000^c^	17 (10 to 25)	5000	0%	Precipitate or exacerbate neuropathy in vitamin B_12_ deficient individuals
Vitamin D^d^ – mcg and Vitamin E^d^ - mg	D: 100; E: 1000^e^	3 (1 to 9)	D: 50; E: 39,545 and 18,000^f^	0%	Vitamin D: Hypercalcemia. Vitamin E: Increased tendency to hemorrhage seen in rats
Calcium - mg	2500	6 (2 to 13)	5000	0%	Hypercalcemia, renal insufficiency
Iron - mg	45	7 (3 to 14)	--	--	--
Magnesium - mg	350^c; g^	6 (2 to 13)	360	6 (2 to 13)	Diarrhea
Zinc - mg	40 mg	6 (2 to 13)	--	--	--
Manganese^d^ - mg	11 mg	8 (4 to 16)	--	--	--
**Vitamins and Minerals (without ULs**^**a **^**and LOAELs**^**b**^**)**					
**Product names**	**% (95% CI)**	**Adverse Events Reported in the Literature**^**h**^			
Pantothenic acid, vitamin B_5_, pantethine, pantothenol, or D-pantothenate and Potassium^i^	37 (28 to 49)	Forms of pantothenic acid: Diarrhea seen with 10 to 20 grams/day of calcium D-pantothenate [28]. Case report of eosinophilic pleuropericardial effusion in an elderly woman taking 10 mg of biotin and 300 mg of pantothenic acid daily for two months [29]. Nausea and heartburn, have been reported with pantethine [30]			
		Potassium: Supplementation of potassium only is generally prescribed to treat hypokalemia while preventing hyperkalemia and medication interactions. Mild effects include nausea, vomiting, abdominal discomfort, and diarrhea [28]			

^a^Tolerable Upper Intake Level.

^b^Lowest Observed Adverse Effect Level: The lowest intake (or experimental oral dose) at which an adverse effect has been identified.

^c^The UL for niacin, folate, and magnesium apply to synthetic forms obtained from supplements, fortified foods, or a combination of the two.

^d^Nutrient analysis software for food intakes of vitamin E, vitamin D and manganese have less than 50% coverage for nutrient; thus prevalence estimates of nutrient intakes exceeding the UL are conservative.

^e^The UL for vitamin E applies to any form of supplemental α-tocopherol, fortified foods, or a combination of the two.

^f^LOAEL for vitamin E is based on 500 mg/kg of α-tocopherol.

^g^The UL for magnesium is based on gastrointestinal effects from consumption of 350 mg or more of the synthetic form.

^h^Based on database searches of MEDLINE, EMBASE, PsychINFO, the Cochrane Library, CINAHL, NAPRALERT, MedEffect™ Canada, International Pharmaceutical Abstracts, CISCOM, and HerbMed.

^i^Potassium in multi-vitamin and mineral supplements are limited to 99 mg per day. Reported frequency here includes only supplementation of potassium as the single nutrient as adverse effects have only been reported with this.

**Table 3:** Descriptions of potential adverse events for non-nutrient based NHPs used by sample based on database search of reported adverse events for each NHP

**NHP used in sample**	**Adverse events reported for that NHP in the literature**^**a**^
Cranberry (*Vaccinium macrocarpon*)	More than one litre daily may cause kidney stones [31]. (Note: In this sample, cranberry pills were used and did not exceed this level)
Dehydroepiandrosterone (DHEA) or 5-Dehydro-epiandrosterone (5-DHEA)	People with mood disorders may experience mania, irritability, and sexual inappropriateness [32-35]
Devil’s Claw (*Harpago-phytum procumbens*)	Mild gastrointestinal upset, hypotension, diarrhea, loss of taste, anorexia, headache, and tinnitus [32-34]. May interact with warfarin; one case report of purpura [36]
Dong Quai (*Angelica sinensis*), Chinese angelica	In a cross-sectional survey (n = 1818), one case was identified as a potential significant interaction between dong quai and anticoagulant/antiplatelet agents [37]
Echinacea (*Echinacea angustifolia, Echinacea pallida, Echinacea purpurea*)	Gastrointestinal upset and rashes; in rare cases, has been associated with allergic reactions that may be severe [38]. May interact with amoxicillin [39]
Evening Primrose Oil (*Oenothera biennis*)	Case reports of seizures in patients with/without known seizure disorders [40,41]. In cross-sectional survey (n = 1818), two cases of potential significant interactions with anticoagulant/antiplatelet agents identified [37]
Feverfew (*Tanacetum parthenium; syn. Chrysanthemum parthenium (L.) Pers., Pyrethrum parthenium Sm.)*	Gastrointestinal upset [42,43], nervousness, insomnia [44], and possible allergic responses in those sensitive to chrysanthemums, daisies, or marigolds. Potential cross-reactivity with other members of the Compositae family [45]. In cross-sectional survey (n = 1818), two cases of potential clinically significant interactions with anticoagulant/antiplatelet agents identified [37].
Flaxseed (common flax, linseed, *Linum usitatissimum*)	Rarely, flaxseed (not oil form) has caused gastrointestinal distress [46-50]. A double-blind placebo-controlled trial suggested there may be increased episodes of mania and hypomania in people with bipolar disorder [46]
Garlic (*Allium sativum*)	Breath and body odour, and allergic reactions [51]. Excess use associated with spontaneous epidural hematoma [52]. Potential reactions include bleeding and hypoglycemia (likely not clinically significant) [53]. In cross-sectional survey (n = 1818), 25 cases of potential clinically significant interactions with anticoagulant/antiplatelet agents identified [37]
Ginkgo (*Ginkgo biloba*)	Surveillance studies (> 10,000 people), found 1.69% incidence of symptoms such as headache and gastrointestinal complaints [54]. Bleeding indicated in a few case reports [55]. May cause allergic hypersensitivity, including Stevens-Johnson syndrome [56-58]. In cross-sectional survey of 1818 patients, 20 cases of potential clinically significant interactions with anticoagulant/antiplatelet agents identified [37]. Infrequent mild gastrointestinal discomfort has been reported when Ginkgo is taken with selective serotonin reuptake inhibitors (SSRIs) [59]. May interact with thiazides [60,61], and nifedipine [62,63]
Ginseng (American, Asian, Chinese, Korean red; *Panax ginseng*, *Panax* spp. including *P. ginseng* and *P. quinquefolius*)	Long-term use of Panax and American ginseng associated with skin rash, itching, diarrhea, sore throat, loss of appetite, excitability, anxiety, depression, or insomnia [53,64]. Few reports of headache, fever, dizziness/vertigo, blood pressure changes, chest pain, difficult menstruation, heart palpitations, leg swelling, nausea, vomiting, manic episodes in bipolar disorder, or Stevens-Johnson syndrome (may have been due to product contaminants) [53]. High intake of American ginseng may result in hypoglycemia in people with/without diabetes [65]. May interact with anticoagulants/antiplatelets [66,67], diabetes medications [37], digoxin [68], estrogenic agents [69-71], furosemide [72], monoaminergic agents [73-75], nifedipine [76]
Ginseng (American, Asian, Chinese, Korean red; *Panax ginseng*, *Panax* spp. including *P. ginseng* and *P. quinquefolius*)	Long-term use of Panax and American ginseng associated with skin rash, itching, diarrhea, sore throat, loss of appetite, excitability, anxiety, depression, or insomnia [53,64]. Few reports of headache, fever, dizziness/vertigo, blood pressure changes, chest pain, difficult menstruation, heart palpitations, leg swelling, nausea, vomiting, manic episodes in bipolar disorder, or Stevens-Johnson syndrome (may have been due to product contaminants) [53]. High intake of American ginseng may result in hypoglycemia in people with/without diabetes [65]. May interact with anticoagulants/antiplatelets [66,67], diabetes medications [37], digoxin [68], estrogenic agents [69-71], furosemide [72], monoaminergic agents [73-75], nifedipine [76]
Melatonin (N-acetyl-5-methoxytryptamine)	May worsen depression and irritability. Sedative medications (CNS depressants) and benzodiazepines interact with melatonin [77]
Omega-3 Fatty Acids, Alpha-Linolenic Acid	Caution indicated for those with diabetes as may increase blood glucose, at risk of bleeding, or with high LDL levels [78-85]. May interact with anticoagulants/antiplatelets [80,86-90]
Valerian (*Valeriana officinalis*)	Mild impairments in concentration, processing, fatigue (less pronounced than with benzodiazepines) [91-95], dizziness, and headache [96,97]. Drug “hangover” and “withdrawal” effect has been reported with high doses [96]. Delirium, ameliorated by benzodiazepines, indicated in one case report [98]. Some develop a “paradoxical reaction” leading to nervousness, and use for longer than 2 months may result in insomnia [53]. Rare reports of hepatotoxicity with some preparations that include valerian [99] but may have been due to other components. May interact with CNS depressants [91,92,100]. In cross-sectional survey (n = 1818), 15 cases of potential clinically significant interactions with sedatives identified [37]. One case of SSRI use and valerian (with alcohol) indicated mental status changes [101]

^a^Based on database searches of MEDLINE, EMBASE, PsychINFO, the Cochrane Library, CINAHL, NAPRALERT, MedEffect™ Canada, International Pharmaceutical Abstracts, CISCOM, and HerbMed for each NHP used in the sample including common and scientific names, synonyms and the primary active constituents of the NHP.

## Discussion

The results of this analysis indicate that there tends to be a higher prevalence of NHP use among persons with mood disorders, particularly males, than in the general population (i.e., BCNS data). A small proportion of the sample exceeded levels deemed to be the upper margin of safety for the general healthy population for 8 nutrients, none of which represented significant harms. Finally, about 38% of survey respondents were potentially exposing themselves to adverse reactions associated with their use of NHPs that did not contain vitamins or minerals.

The use of NHPs appears to be of a similar scale to that found in other investigations of mental health populations where reported prevalence ranges from 50 to 80% [4]. The common use of NHPs may be attributable to many factors that include media interest in the topic, increased availability of various information sources (e.g., the Internet), the perceived efficacy and “naturalness” of the therapies, desire to reduce side effects, and dissatisfaction with conventional therapies leading to experimentation with different products [102-104]. Unlike other studies, our results did not indicate differences in NHP use according to socioeconomic status which is contrary to the inverse supplement hypothesis [105], which suggests that those in need of more nutrients due to factors such as disease risk or limited income are usually not the ones who take supplements. People with mood disorders may not consider income a barrier if they believe that the NHPs will alleviate symptoms.

The potential health risk associated with intakes above the LOAELs (i.e., skin flushing, mild diarrhea) in a few of the participants is relatively minor. Niacin intake excesses may be due to people receiving pharmacological doses (i.e., 1 to 3 grams daily) to manage serum cholesterol levels as per Canadian lipid guidelines [55]; at least 31 participants had high blood cholesterol levels. We previously reported in this same sample that there was a pattern of correlations between some nutrient intakes (even above the ULs) and overall mental health based on GAF scores [106], suggesting that these individuals may have been benefiting from the additional nutrients. We also note that the *DRI*s are based on healthy populations and may have limited applicability to persons with mental health conditions. For example, lithium in pharmacological doses would certainly be contraindicated in healthy populations, but in individuals with bipolar disorder higher amounts of this mineral are standard treatment. While there was potential for more severe adverse reactions from non-nutrient based NHPs, some of these products are also used as sources for conventional drugs [52].

The modest sample size may be interpreted as a limitation. However, unlike the large population surveys on NHP use, our study collected detailed data (e.g., types, dose, frequency, nutrients from food plus supplements), verified mental health diagnosis, and conducted an assessment of potential for adverse events. The sample was comprised of mainly females, individuals with a mental health condition that were generally high functioning, and resided in an urban region which may limit the generalizability of findings. In addition, it cannot be determined how well MDABC membership reflects the population of all individuals with mood disorders. The prevalence of NHP use in this study may be overstated as the region in which the data was collected is reported to have a higher proportion of alternative medicine practices compared to other Canadian provinces [10]. The prevalence estimate for potential adverse effects may be conservative as data is lacking about interactions of NHPs with each other and with foods, and many surveillance systems are passive and contain incomplete data (e.g., a specific dose level that led to an adverse effect). Conversely, the potential for adverse events may be overstated as the information obtained tended to be based on anecdotal reports and not prospective data that specifically documents harms.

## Conclusions

Since NHP use among individuals with mood disorders is prevalent, health care providers may best serve these clients by becoming more knowledgeable about their characteristics. Further research on the safety of NHPs including their impact on the course and prognosis of mental health conditions is also of relevance.

## Abbreviations

BCNS: British Columbia Nutrition Survey; CI: Confidence Interval; LOAEL: Lowest Observed Adverse Effect Level; NHP: Natural Health Product; mcg: Micrograms; mg: Milligrams; UL: Tolerable Upper Intake Level

## Competing interests

The authors declare that they have no competing interests.

## Authors’ contributions

KMD and BJK designed the study and planned its coordination and data collection. KMD carried out the data collection as part of her PhD dissertation, under the supervision of BJK. Both authors worked on writing the manuscript. Both authors read and approved the final manuscript.

## Authors’ information

KMD carried out this study as part of her requirement for a PhD in the Faculty of Medicine, University of Calgary, under the supervision of BJK, and is currently a postdoctoral research fellow (Chronic Condition Self-Management) in the School of Population and Public Health at the University of British Columbia. BJK is a Professor in the Faculty of Medicine at the University of Calgary who studies nutrition in relation to mental development and function.

## Pre-publication history

The pre-publication history for this paper can be accessed here:

http://www.biomedcentral.com/1472-6882/13/80/prepub
